# Color-induced cognitive conflicts affect muscle activity prior to gait initiation in the Go/No-go task

**DOI:** 10.3389/fnhum.2024.1463220

**Published:** 2024-10-02

**Authors:** Takayuki Horinouchi, Haruki Ishida, Kangjing Yang, Jingnan Li, Takuya Morishita, Tatsunori Watanabe, Hikari Kirimoto

**Affiliations:** ^1^Department of Sensorimotor Neuroscience, Graduate School of Biomedical and Health Sciences, Hiroshima University, Hiroshima, Japan; ^2^Japan Society for the Promotion of Science, Tokyo, Japan; ^3^Department of Rehabilitation, Kurashiki Rehabilitation Hospital, Okayama, Japan; ^4^Defitech Chair of Clinical Neuroengineering, Neuro X Institute (INX), École Polytechnique Fédérale de Lausanne (EPFL), Campus Biotech, Geneva, Switzerland; ^5^Defitech Chair of Clinical Neuroengineering, INX, EPFL Valais, Clinique Romande de Réadaptation, Sion, Switzerland; ^6^Faculty of Health Sciences, Aomori University of Health and Welfare, Aomori, Japan

**Keywords:** Go/No-go task, prior knowledge of color, gait initiation, tibialis anterior muscle, center of pressure

## Abstract

**Introduction:**

In traffic rule, green/blue means go, and red means stop. It has been shown that this prior knowledge about traffic signal colors can affect reaction times (RTs). For example, RTs are longer when responding to a red “Go” signal and withholding the response to a blue “No-go” signal (Red Go/Blue No-go task) than when responding to a blue “Go” signal and withholding the response to a red “No-go” signal (Blue Go/Red No-go task), when responses are provided by button press. However, it remains unknown whether this holds in different actions. The aim of this study was to investigate the effect of prior knowledge of color on gait initiation in a Go/No-go task.

**Methods:**

Seventeen participants performed Green Go/Red No-go and Red Go/Green No-go tasks, in which they stepped forward from a force plate in response to a green or red signal and withhold the response to red or green signal, respectively. We recorded the center of pressure (COP) and electromyogram (EMG) from the bilateral tibialis anterior muscles during gait initiation.

**Results:**

The onset of COP movement and toe-off time as well as COP displacements did not differ between the Go/No-go tasks. The EMG onset for the stance leg was delayed in the Red Go/Green No-go than Green Go/Red No-go task.

**Discussion:**

These findings suggest that the conflict between prior knowledge of color related to traffic rule and the meaning of the stimulus color affects muscle activity but not COP characteristics during gait initiation, highlighting two distinct motor control mechanisms, where the initial phase is influenced by cognitive load while the subsequent phase remains unaffected. This dissociation suggests that the later phase of gait initiation relies on robust spinal loops and central pattern generators, which are less influenced by cognitive factors such as prior knowledge.

## 1 Introduction

In our daily lives, we encounter situations where we make decisions to either execute or inhibit an action based on color. For example, after detecting the color of traffic lights, we go forward when the lights are blue/green and stop when the lights are red, based on an international standard for the meaning of colors for safety signs. Although simple reaction time (RT) to a signal has been demonstrated to be similar between red and blue lights (Eason et al., 1967), a previous study reported that button-press RTs were prolonged when the color of pedestrian traffic signal was reversed, even though reversing the shape did not change the RTs (Peschke et al., 2013). In other words, pedestrians tend to make decisions primarily based on color rather than shape when responding to these signals. Therefore, it is important to understand how the meaning of color affects our actions possibly to prevent traffic accidents.

In the past few years, we have investigated how the meaning of color influenced RTs using a Go/No-go task, in which participants are required to respond when a target (Go) signal is presented but to refrain from responding when a non-target (No-go) signal is presented. Our findings indicated that RTs were longer when responding to a red “Go” signal and withholding the response to a blue “No-go” signal (Red Go/Blue No-go task) than when responding to a blue “Go” signal and withholding the response to a red “No-go” signal (Blue Go/Red No-go task) (Kubo et al., 2021). This may reflect that the processes of perceiving visual information and executing movements are subconsciously affected by the traffic rule. In the same study, we also confirmed that N2 amplitude of event-related potentials (ERPs) at the front-central site (Fz and Cz), which reflects a conflict detection (Boenke et al., 2009; Botvinick et al., 2001; Pan et al., 2016), was larger in the Red Go/Blue No-go than Blue Go/Red No-go task (Kubo et al., 2021). Furthermore, our following study revealed that frontal beta desynchronization, which can reflect the facilitation of motor execution, was increased when responding to a blue signal, and that the onset of frontal theta oscillation was delayed during the Red Go/Blue No-go task (Horinouchi et al., 2023). On the other hand, in addition to a finding of simple RT to a blue light being similar to that to a red light (Eason et al., 1967; Horinouchi et al., 2022, 2023), the latency of C1 component of the ERP observed at the occipital site (Oz), representing the activity of the primary visual cortex, did not differ between the Blue Go/Red No-go and Red Go/Blue No-go tasks (Kubo et al., 2021). In other words, the difference in RT between these Go/No-go tasks is not driven by a difference in color itself or the initial visual processing of lights. Instead, we interpret that this difference in RT is due to a conflict between the prior knowledge of color about traffic rules acquired in daily life and the meaning of the presented stimulus, similar to so-called the Stroop effect (Stroop, 1935).

Although previous studies collectively indicate the effect of meaning of color on RTs, experiments in these studies were conducted using button-press responses only, leaving the possibility that the effect could depend on the response type. Gait initiation is an important part of locomotion and a repetitive daily task; therefore, investigating the effects on this particular task could enhance our understanding of this phenomenon and its practical applications. During gait initiation, the center of pressure (COP) initially shifts to the swing leg side and backward, then to the side of the stance leg, and finally progresses forward (Winter, 1995). Analysis of the COP trajectory enables an identification of a time point of movement onset, a time point of the greatest displacement of COP towards the swing leg, and a time point at which a toe of the swing leg is off the ground (Halliday et al., 1998; Mickelborough et al., 2004; Palmisano et al., 2022). Recording the electromyogram (EMG) further enables to analyze muscle activities that generate the COP trajectory. Specifically, prior to gait initiation, there is a decrease in activity of the soleus muscles and an increase in activity of the tibialis anterior (TA) muscles and the hip abductor muscles (Delafontaine et al., 2019; Kirker et al., 2000; Mickelborough et al., 2004), and these muscle activities induce the initial COP shifts to the swing leg side and backward, known as an anticipatory postural adjustment (APA) (Brunt et al., 1991; Palmisano et al., 2022; Winter, 1995). In a number of previous studies, a response conflict induced by stimulus-response compatibility tasks has been confirmed to affect APAs associated with gait initiation (Watanabe et al., 2015, 2016, 2017). In particular, EMG onset of the TA muscle of the stance leg has been shown to delay during gait initiation performed concurrently with the Stroop task (Fernandes et al., 2016). Therefore, it is reasonable to hypothesize that gait initiation is affected by subconscious conflict induced by the difference between the prior knowledge of color and the meaning of the presented stimulus.

The aim of the present study was to investigate the effect of prior knowledge of color about traffic rules on gait initiation in a Go/No-go task. Although we have used a blue LED in previous studies (Horinouchi et al., 2022, 2023; Kubo et al., 2021), we adopted a green LED in the present study because it is closer to the color of traffic lights. To achieve our aim, we evaluated EMG of the TA muscles and COP during gait initiation using two types of simple reaction and Go/No-go tasks: a Green simple reaction task, a Red simple reaction task, a Green Go/Red No-go task, and a Red Go/Green No-go task. We hypothesized that onsets associated with COP trajectory and the TA muscle activity would be later in the Red Go/Green No-go than Green Go/Red No-go task but not in the simple reaction task.

## 2 Materials and methods

### 2.1 Participants

To determine the minimum sample size required for this study, a statistical power analysis was performed using G*Power software version 3.1.9.7 (Kiel University, Kiel, Germany). The analysis revealed that a minimum sample size of 13 participants was required for a paired *t*-test, with an effect size of 0.85, a power of 0.8, and a significance level of 0.05. The effect size was determined based on a previous study using the button-pressing Go/No-go task (Horinouchi et al., 2023). Based on this calculation, 17 healthy participants (6 females, mean age ± SD = 24.2 ± 3.1 years) were recruited to participate in this study. All participants had normal or corrected-to-normal vision. Written informed consent was obtained from all participants before beginning the experiment, which was performed according to the principles of the Declaration of Helsinki. The study was approved by the Ethics Committee for Clinical Research of Hiroshima University (No. C-242).

### 2.2 Design and procedure

**Figure 1:**
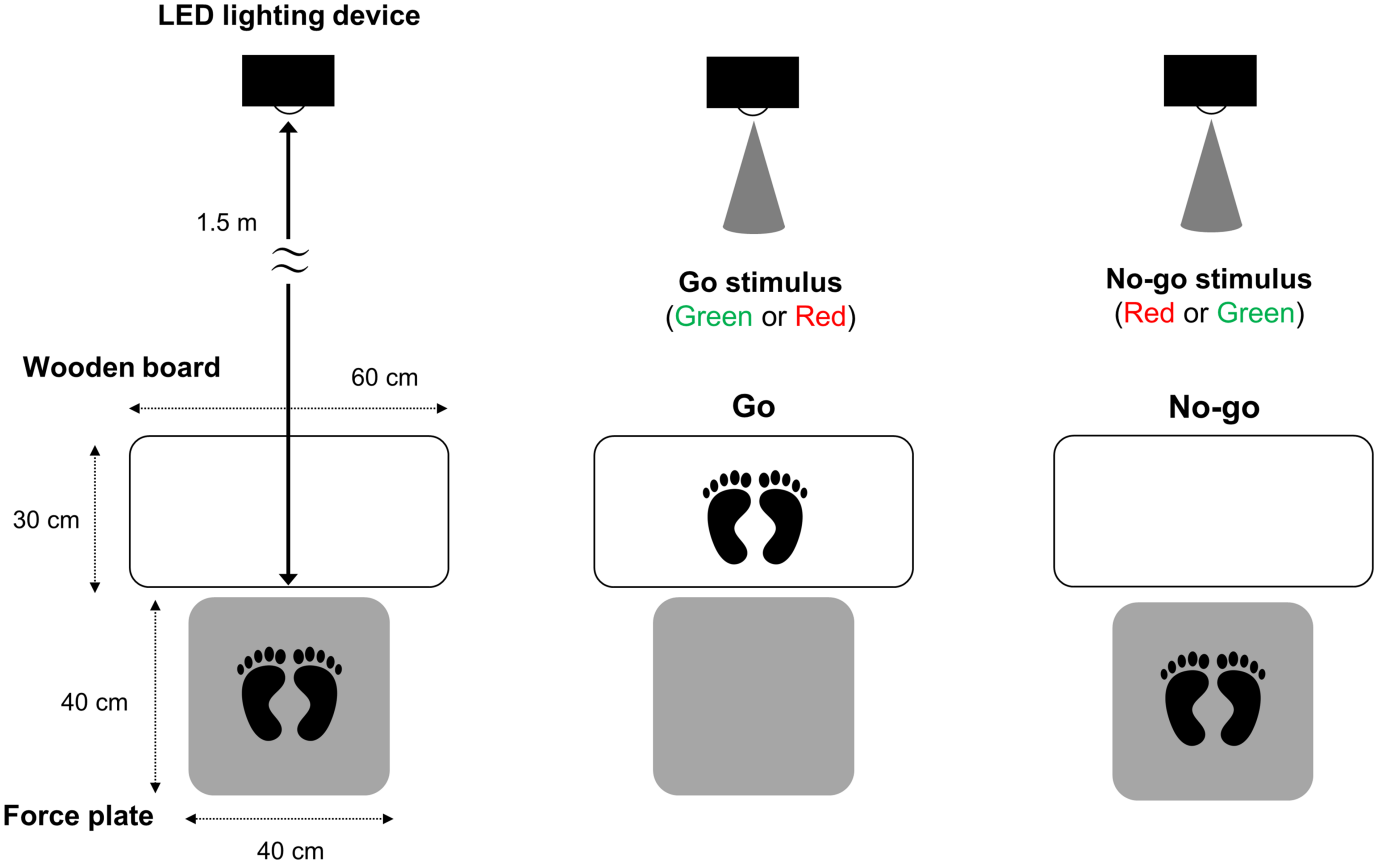
Schematic illustration of the experiment participants stood on a force plate 1.5 m in front of the LED lighting device and stepped forward in response to the go stimulus and withheld the response for the no-go stimulus.

Two types of simple reaction and Go/No-go tasks were performed in a random order: a Green simple reaction task, a Red simple reaction task, a Green Go/Red No-go task, and a Red Go/Green No-go task (Figure 1). In all tasks, participants maintained an upright stance at the center of a 40 cm square force plate (CFP400PA102RS, Leptrino, Nagano, Japan), evenly distributing their weight across both feet. A wooden board (30 cm length × 60 cm width) of the equal height to the force plate was placed in front of it. It was confirmed that the legs did not get out of the wooden board when participants stepped naturally. Signals (green and red lights) were presented using a custom-made LED lighting device (4Assist, Tokyo, Japan) (Watanabe et al., 2021), which was placed 1.5 m in front of the participants at eye level. Green and red signals were confirmed to be at the same illuminance level (250 cd/m^2^), using a luminance meter (HD2302.01, Delta OHM, Padova, Italy). To eliminate a confounding factor related to the visual stimulus location (Mansfield, 1973; Rains, 1963), green and red LED bulbs were placed close to each other, and these lights were visible from one spot through a hole (5 mm) created in the device (Horinouchi et al., 2022).

#### 2.2.1 Simple reaction task

Simple reaction tasks were conducted to examine the effect of color itself (green and red) on COP trajectory and EMG activity of the TA muscles. In both Green and Red simple reaction tasks, signals with a duration of 100 ms were presented a total of 10 times. These signals were randomly presented at intervals of 1,000 ± 100 ms following the warning cue, with a 10 s interval between the warning cues. The participants were instructed to step forward onto the wooden board as fast as possible in response to the signal. The leg used to step forward, either left or right, was predetermined for each participant based on their gait initiation in daily life, and it was used for all the tasks. As a result, 2 out of 17 participants stepped forward with the left leg. After each stepping movement, the participants promptly returned to the center of the force plate and subsequently looked at a display below the LED lighting device that provided COP feedback in order to adjust their position to ensure the equal distribution of weight across both feet. Then, the participants stabilized their posture and focused on the LED lighting device.

#### 2.2.2 Go/No-go task

In the Green Go/Red No-go task, green and red signals served as target (Go) and non-target (No-go) signals, respectively. In the Red Go/Green No-go task, red and green signals served as target (Go) and non-target (No-go) signals, respectively. The signals with a duration of 100 ms were presented a total of 30 times. These signals were randomly presented at intervals of 1,000 ± 100 ms following the warning cue, with a 10 s interval between the warning cues. The Go probability for both tasks was set to 1/3 (10 times in each task). The participants were instructed to step forward onto the wooden board as fast as possible in response to Go signals and to withhold the response when a No-go signal appeared (Figure 1). After each stepping movement, participants promptly returned to the center of the force plate and subsequently looked at a display below the LED lighting device that provided COP feedback in order to adjust their position to ensure the equal distribution of weight across both feet. Then, the participants stabilized their posture and focused on the LED lighting device.

### 2.3 Data acquisition and analysis

During both the simple reaction and Go/No-go tasks, the EMG and COP data were sampled along with signals from the LED lighting device using an analog-to-digital converter (PowerLab, AD Instruments, New South Wales, Australia). They were stored on a personal computer for offline analysis (LabChart, AD Instruments, New South Wales, Australia).

#### 2.3.1 EMG data

**Figure 2:**
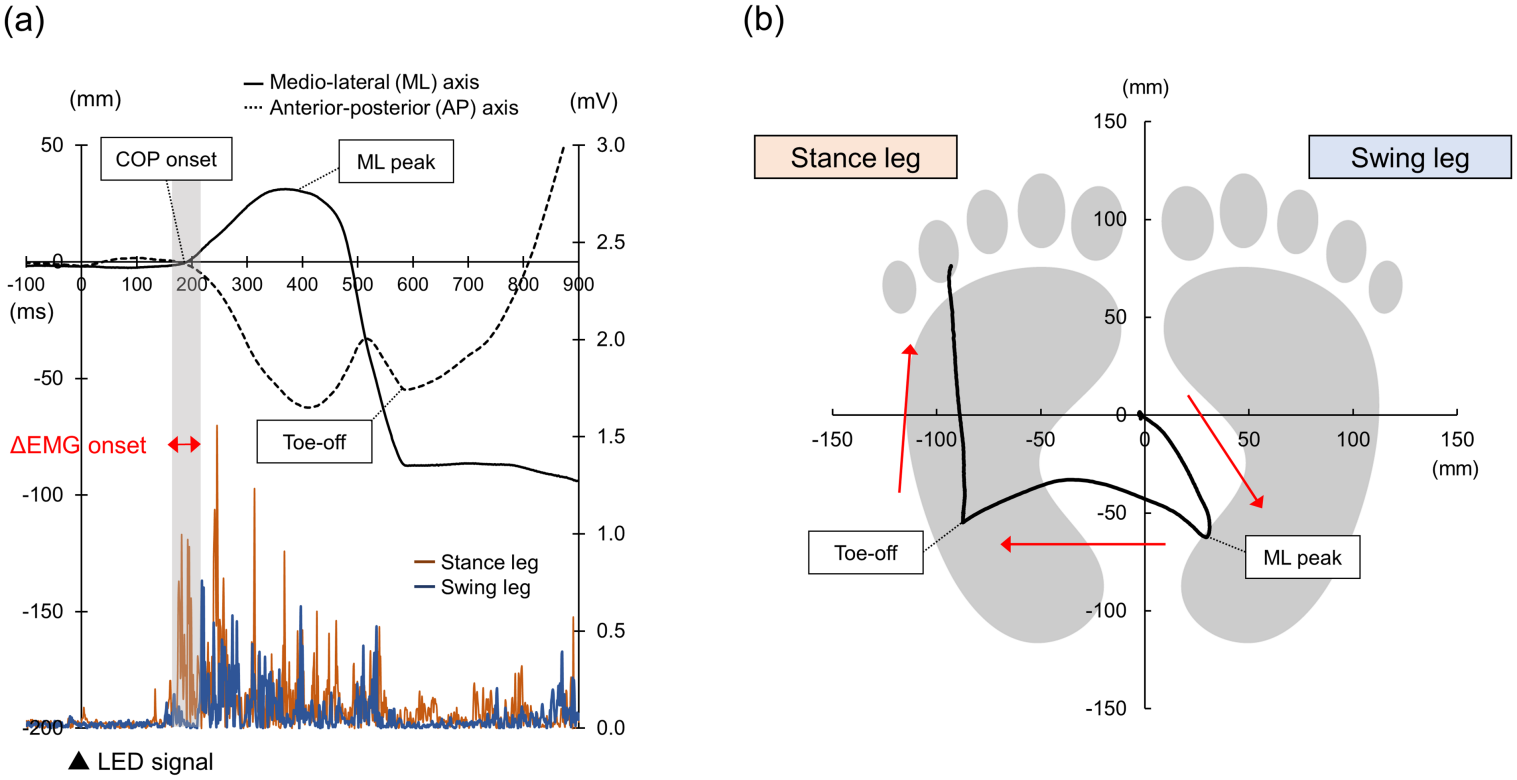
Analysis of EMG and COP data time series data for each COP (medio-lateral and anterior-posterior) axis and EMG of the bilateral TA muscles (a). COP trajectory on a two-dimensional plane (b).

Surface EMG signals were recorded from the right and left TA muscles with disposable Ag/AgCl electrodes. They were amplified (×100) and band-pass filtered at 20–450 Hz with an EMG amplifier system (FA-DL-140, 4Assist), digitized at 1,000 Hz (PowerLab, AD Instruments, New South Wales, Australia), and stored on the personal computer for offline analysis. The EMG signals were rectified and then averaged over a 1,000-ms period prior to the visual stimulus to establish the baseline. EMG onset, which is the premotor time, was defined using MATLAB (MathWorks, Massachusetts, United States) as the first time point at which the EMG signal exceeded the baseline +2SD for at least 10 ms. We also computed the time difference between EMG onset of the stance leg and that of the swing leg and defined it as ΔEMG onset (Tsuru et al., 2020) (Figure 2a). Trials in which the EMG onset was less than 100 ms and trials in which the participants did not respond to Go signals (Go omission errors) or in which participants responded to No-go signals (No-go commission errors) were excluded from the analysis. Go omission errors were defined as trials where no response (EMG signal exceeding the baseline +2SD for at least 10 ms) to the Go signal was observed within 1,000 ms after the signal. No-go commission errors were defined as trials in which a response (EMG signal exceeding the baseline +2SD for at least 10 ms) to the No-go signal was observed within 1,000 ms after the signal. The number of Go omission and No-go commission errors were compared between two Go/No-go tasks.

#### 2.3.2 COP data

Signals from the force plate were sampled at 1,200 Hz, low-pass filtered (20 Hz), and stored on the personal computer for off-line analysis. From the COP trajectories calculated using a custom software (BSMLGR, Leptrino, Nagano, Japan), we identified a time point of movement onset (COP onset), a time point of maximum medio-lateral (ML) displacement of the COP towards the swing leg (ML peak), and a time point at which the swing leg is off the ground (Toe-off), and calculated duration from the visual stimulus to each time point using MATLAB (Figure 2b). The COP onset was defined as the first time point at which the ML COP displacement exceeded +3SD from the pre-stimulus baseline average (−1,000 to 0 ms). The ML peak was defined as a point at which the ML COP displacement reached its maximum. The Toe-off was defined as a point at which the COP started to move forward after moving to the stance leg. Trials in which the COP onset was less than 100 ms were excluded from the analysis. The ML COP displacement at the ML peak was further calculated by subtracting the ML COP value at the COP onset from that at the ML peak. The AP COP displacement at the Toe-off was calculated by subtracting the AP COP value at the COP onset from that at the Toe-off.

### 2.4 Statistical analysis

SPSS Statistics software version 21 (IBM Corporation, NY, United States) was used for statistical analysis. The distribution of data was examined using the Shapiro–Wilk test. If the data were not normally distributed, nonparametric tests were used. Significant level was set at *p* < 0.05.

The independent variable in the simple reaction task was the signal color (green or red), while the dependent variables included EMG data (EMG onset for the stance and swing legs and ΔEMG onset) and COP data (COP onset, ML peak, Toe-off, ML COP displacement, and AP COP displacement). In the Go/No-go task, the independent variable was the combination of signal color and Go/No-go conditions (Green Go/Red No-go and Red Go/Green No-go tasks). The dependent variables in this task included EMG data (EMG onset for the stance and swing legs and ΔEMG onset), COP data (COP onset, ML peak, Toe-off, ML COP displacement, and AP COP displacement), as well as the number of errors (Go omission errors and No-go commission errors).

#### 2.4.1 Simple reaction task

Paired *t*-tests were used to compare EMG onsets (stance and swing leg), ΔEMG onset, time points of COP onset, ML peak, and Toe-off, and COP displacements at the ML peak and Toe-off between the Green and Red simple reaction tasks.

#### 2.4.2 Go/No-go task

Paired *t*-test was used to compare EMG onsets (stance and swing leg), ΔEMG onset, time points of COP onset, ML peak, and Toe-off, and COP displacements at the ML peak and Toe-off between the Green Go/Red No-go and Red Go/Green No-go tasks.

A Wilcoxon signed-rank test was used to compare the number of errors between the Green Go/Red No-go and Red Go/Green No-go tasks.

## 3 Results

### 3.1 Simple reaction task

**Figure 3:**
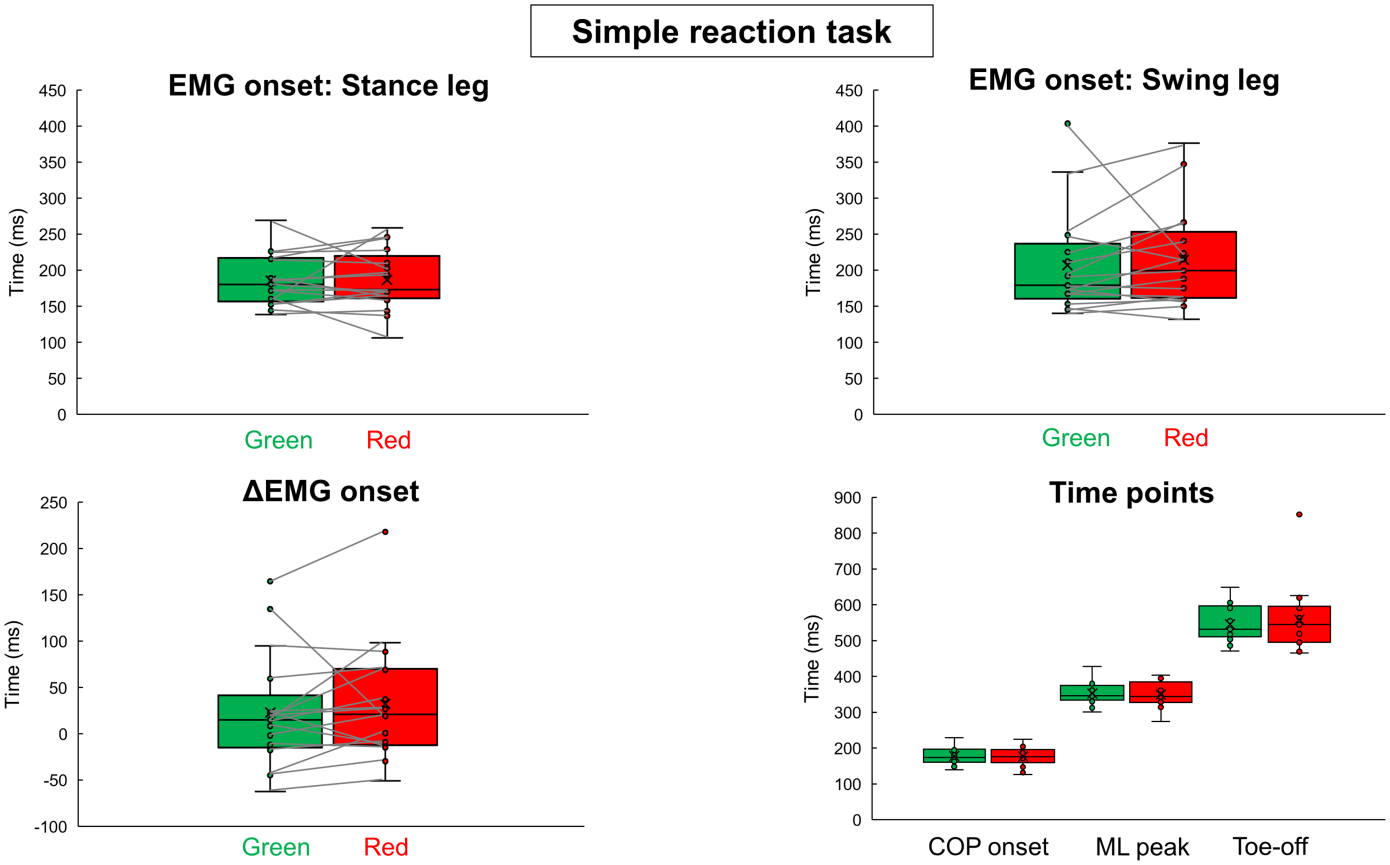
EMG onsets, ΔEMG onset, and time points calculated from COP trajectory in the simple reaction tasks results of the green simple reaction task and the red simple reaction task are shown in box-and-whisker plots. The small circles indicate the values for each participant. The crossed symbols indicate the mean values.

Table 1 shows all results in the simple reaction tasks. Figure 3 shows results of EMG onsets, ΔEMG onset, and time points of COP onset, ML peak, and Toe-off in the simple reaction tasks. There were no significant differences between the signal colors (green and red) in all the variables calculated from the EMG and COP data.

**Table 1:** EMG and COP results in the simple response tasks.

Simple reaction task							
		Green (mean ± SE)	Red (mean ± SE)	Paired *t*-test			*p*-value
				df	*T*-value	Effect size (Cohen’s *d*)	
EMG onset (ms)	Swing leg	207 ± 17	214 ± 17	16	−0.53	0.11	0.60
	Stance leg	185 ± 9	187 ± 10	16	−0.14	0.03	0.89
ΔEMG onset (ms)		23 ± 15	32 ± 15	16	−0.88	0.15	0.39
COP onset	Time (ms)	178 ± 6	176 ± 7	16	0.32	0.07	0.76
ML peak	Time (ms)	352 ± 7	349 ± 9	16	0.56	0.11	0.60
	Displacement (mm)	49 ± 6	51 ± 6	16	−0.38	0.07	0.69
Toe-off	Time (ms)	545 ± 12	559 ± 22	16	−0.74	0.18	0.47
	Displacement (mm)	53 ± 5	52 ± 6	16	0.47	0.06	0.62

SE, standard error; df, degree of freedom.

### 3.2 Go/No-go task

**Figure 4:**
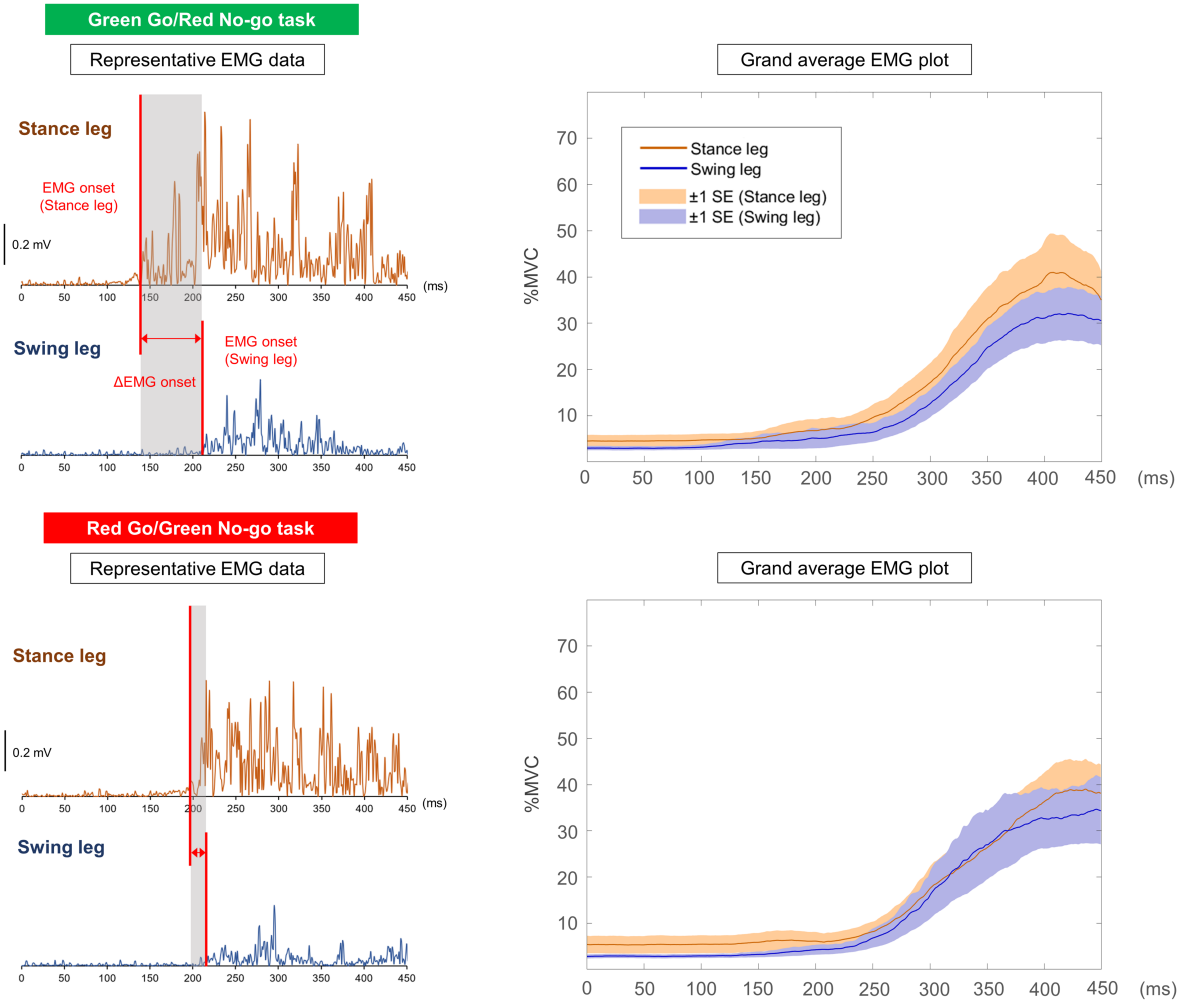
Representative EMG data and grand average EMG plots in Go/No-go tasks the timing of signal presentation is represented as 0 ms. In the grand average EMG plots, shaded areas indicate ±1 SE.

**Figure 5:**
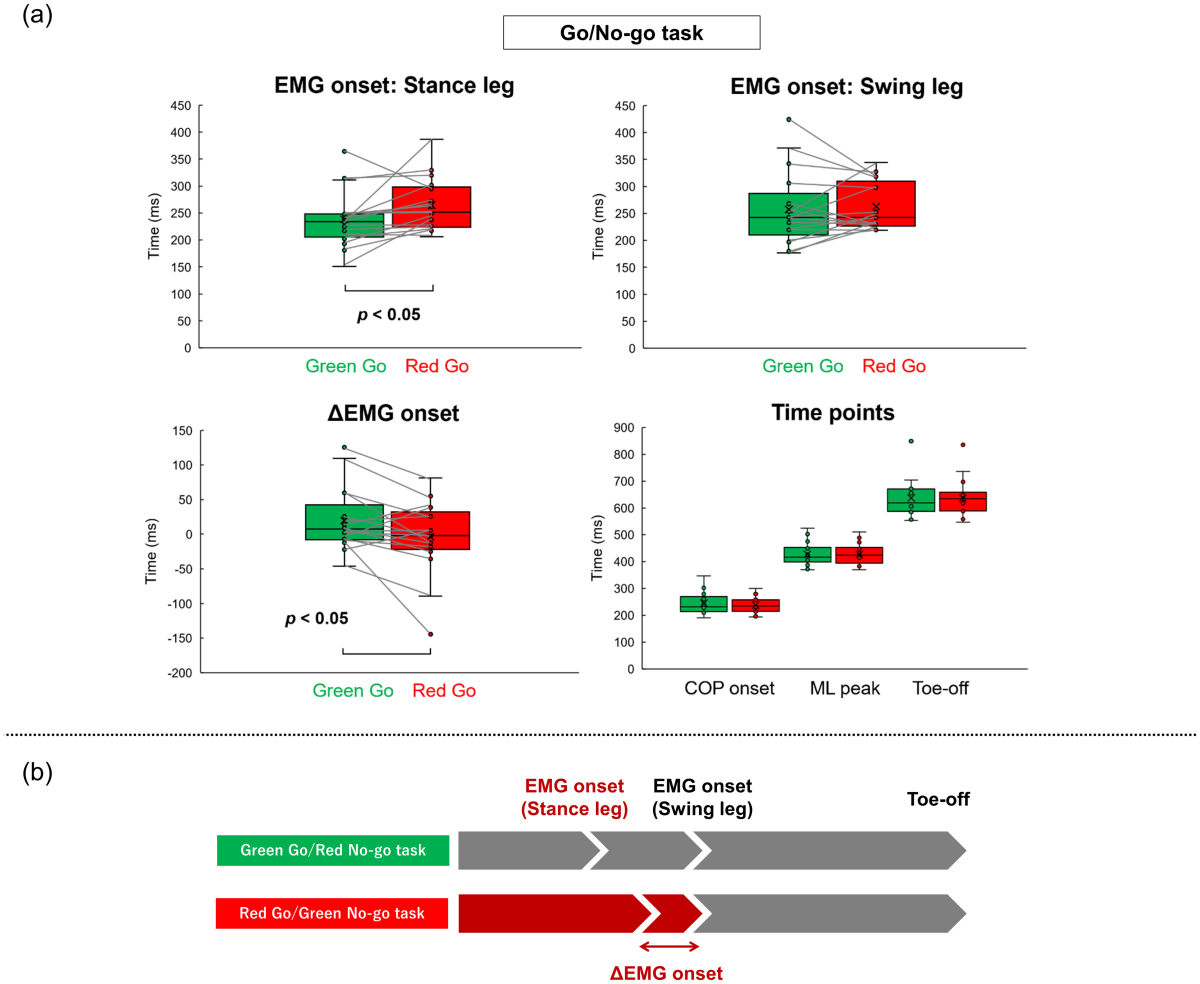
Summary of results in Go/No-go tasks result of EMG onsets, ΔEMG onset, and time points calculated from COP trajectory in the Go/No-go tasks (a). Conceptual diagram of results in the Go/No-go tasks (b).

Table 2 shows all results in the Go/No-go tasks. Figure 4 shows the representative EMG data and grand average EMG plots in the Go/No-go tasks. Figure 5a shows results of EMG onsets, ΔEMG onset, and time points of COP onset, ML peak, and Toe-off in the Go/No-go tasks. A paired *t*-test revealed that EMG onset of the stance leg was significantly delayed when responding to a red signal (Red Go/Green No-go task) as compared with when responding to a green signal (Green Go/Red No-go task) (df = 16, *T* = −2.31, *p* = 0.03). Furthermore, a paired *t*-test revealed that the ΔEMG onset was significantly shorter when responding to a red signal (Red Go/Green No-go task) as compared with when responding to a green signal (Green Go/Red No-go task) (df = 16, *T* = 2.31, *p* = 0.03). On the other hand, there was no significant difference in EMG onset of the swing leg. There were no significant differences in time points of COP onset, ML peak, and Toe-off, or COP displacements.

**Table 2:** EMG and COP results in the Go/No-go tasks.

Go/No-go task									
		Green Go/Red No-go (mean ± SE)	Red Go/Green No-go (mean ± SE)	Paired *t*-test			WSR test		*p*-value
				df	*T*-value	Effect size (Cohen’s *d*)	*W*	Effect size (*r*)	
EMG onset (ms)	Swing leg	257 ± 16	262 ± 11	16	−0.44	0.09	—	—	0.67
	Stance leg	239 ± 13	265 ± 12	16	−2.31	0.52	—	—	0.03^*^
ΔEMG onset (ms)		20 ± 11	−4 ± 13	16	2.31	0.47	—	—	0.03^*^
Number of errors: median (IQR)	Go omission	3 (0–4)	1 (0–4)	—	—	—	41	0.05	0.87
	No-go commission	0 (0–0)	0 (0–1)	—	—	—	11	−0.19	0.61
COP onset	Time (ms)	244 ± 10	237 ± 8	16	1.14	0.19	—	—	0.26
ML peak	Time (ms)	427 ± 10	427 ± 10	16	−0.05	<0.01	—	—	0.98
	Displacement (mm)	60 ± 6	61 ± 4	16	−0.47	0.07	—	—	0.63
Toe-off	Time (ms)	638 ± 17	636 ± 17	16	0.25	0.02	—	—	0.85
	Displacement (mm)	60 ± 5	60 ± 5	16	0.65	<0.01	—	—	0.99

SE, standard error; df, degree of freedom. WSR test, Wilcoxon signed-rank test; IQR, interquartile range.

## 4 Discussion

The present study aimed to investigate the effect of prior knowledge of color about traffic rules on gait initiation in the Go/No-go task. For that purpose, we evaluated EMG of the TA muscles and COP during gait initiation using two types of simple reaction and Go/No-go tasks: a Green simple reaction task, a Red simple reaction task, a Green Go/Red No-go task, and a Red Go/Green No-go task. As a result, in the simple reaction task there were no significant differences in all the variables between the two different signal colors. Additionally, in the Go/No-go tasks, there was no significant difference in the swing leg EMG onset. Time points of COP onset, ML peak, and Toe-off did not differ significantly between the two Go/No-go tasks. On the other hand, the stance leg EMG onset was significantly delayed in the Red Go/Green No-go task compared with the Green Go/Red No-go task. Furthermore, the ΔEMG onset was significantly shorter in the Red Go/Green No-go task compared with the Green Go/Red No-go task (Figure 5b).

Similar to previous studies using button-press responses (Eason et al., 1967; Horinouchi et al., 2022), there was no effect of signal color on gait initiation in the simple reaction task. This finding suggests that the perceptual processing is similar between the green and red colors even in gait initiation. Our previous studies have confirmed that there was no difference in the latency of C1 component of ERPs observed at the occipital site (Oz) (Kubo et al., 2021) or in occipital brain oscillations (Horinouchi et al., 2023). These neurophysiological findings also suggest that color itself does not affect simple RTs. Thus, it is plausible to assume that the difference in EMG onset of the TA muscle observed in the Go/No-go task was not due to a difference in color itself, but rather to a conflict between the semantic context of color and the meaning of the presented stimulus.

The mean EMG onset of the stance leg was significantly delayed in the Red Go/Green No-go task compared with the Green Go/Red No-go task. The premotor time prolongation in the Red Go/Green No-go task was approximately 25 ms, which is similar to previous studies using button-press responses (Horinouchi et al., 2022, 2023; Kubo et al., 2021). Similarly, in a previous study using a stop-signal task, the stop-signal RT, which reflects the time taken to cancel a response after its initiation, has been reported to be approximately 25 ms shorter when the stop stimulus was presented in red than green color (Blizzard et al., 2017). In addition, it has been demonstrated that red color evokes avoidance motivation while green colors evoke approach motivation (Bouhassoun et al., 2022). From these studies, it can be suggested that green colors promote execution whereas red color promotes inhibition, which may be related to traffic rules. Therefore, it is likely that the cognitive load induced by a conflict between the prior knowledge of color and the meaning of the presented stimulus delayed the generation of motor commands during gait initiation.

On the other hand, the mean EMG onset of the swing leg did not differ between the two Go/No-go tasks, and the mean ΔEMG onset was significantly shorter in the Red Go/Green No-go task compared with the Green Go/Red No-go task. These results are consistent with a previous study in which EMG onset of the stance leg TA muscle, but not of the swing leg TA muscle, was delayed during gait initiation performed concurrently with the Stroop task (Fernandes et al., 2016). The stance leg TA muscle is generally active prior to the swing leg TA muscle during gait initiation, as reported in previous studies (Brunt et al., 1991; Fortin et al., 2015; Mickelborough et al., 2004). Therefore, our findings indicate that a conflict between the prior knowledge of color and the meaning of the presented stimulus impacts only the initial motor command sent to the stance leg TA muscle and not the accompanying motor mechanisms. Additionally, while factors such as aging and the method of presenting Go signals can influence EMG and COP displacement during the initial phase of gait initiation (Chen et al., 2023; Tard et al., 2016; Wang et al., 2017), subsequent gait initiation intervals often remain unaffected due to the stable mechanisms provided by spinal peripheral loops (Brunt et al., 1999; Henriksson and Hirschfeld, 2005; Kukulka et al., 2009). This is consistent with research indicating that some motor programs involved in gait initiation are influenced by cognitive demands, while the others, regulated by stable neural circuits, are not (Brunt et al., 1999). Therefore, it might be that humans are able to adjust ΔEMG onset in order to compensate for the delay in the initial command. This adaptive and compensatory mechanism may be attributed to the presence of distinct motor programs in the initial and later phases, with cognitive load, like the prior knowledge of color, affecting only the initial phase, such as APAs, and not the subsequent phase controlled predominantly by spinal circuits and central pattern generators.

Time points of COP onset, ML peak, and Toe-off did not differ significantly between the two Go/No-go tasks. Neither did the ML COP displacement at the ML peak and the AP COP displacement at the Toe-off. These results suggest that the prolonged premotor time due to the conflict between the prior knowledge of color and the meaning of the presented stimulus may not necessarily affect COP characteristics during gait initiation. However, previous studies have consistently shown that cognitive load affects COP during gait initiation (Watanabe et al., 2015, 2016, 2017). For example, COP onset during gait initiation has been reported to be later when responding to incongruent than congruent stimuli (Gélat et al., 2011). Furthermore, COP onset during a choice reaction stepping task was delayed when a warning cue provided incorrect information (invalid cue) (Tard et al., 2016). In the present study, the premotor time for the swing leg was not affected by the stimulus color in the Go/No-go tasks; therefore, it appears, as mentioned above, that the conflict between the prior knowledge of color and the meaning of the presented stimulus influences only the initial motor command sent to the stance leg TA muscle and not the accompanying motor mechanisms, including the COP characteristics. This result is somewhat inconsistent with previous studies in which the stimulus color affected the button-press RTs in the Go/No-go tasks (Horinouchi et al., 2022, 2023; Kubo et al., 2021). While visuomotor transformations during button-press responses to stimuli are processed mainly in the cortex, gait initiation is controlled not only by the cortex but also by the subcortical area and the spinal cord (Demain et al., 2014; Koenraadt et al., 2014; Queralt et al., 2010; Takakusaki et al., 2016). In particular, APAs have been proposed to be generated by signals from the supplementary motor area or the premotor cortex via the pedunculopontine nucleus (Takakusaki et al., 2003, 2016). Therefore, these differences in the underlying neurophysiological processes between the button-press response and gait initiation movement might have driven our findings. Specifically, unlike button-pressing tasks in previous studies (Horinouchi et al., 2022, 2023; Kubo et al., 2021), the central nervous system only triggers the subcortical mechanisms that drive the gait initiation (Takakusaki et al., 2003, 2016). Furthermore, as the EMG onset of the stance leg TA was delayed while that of the swing leg TA was not, it is possible that distinct motor programs in the initial and subsequent phases of gait initiation contributed to our findings, as stated previously. Therefore, it is reasonable that while the EMG onset of the initial stance leg TA was delayed, the COP characteristics, which reflect the subsequent gait initiation controlled by spinal circuits and CPGs, remained unchanged. Future research should aim to reinforce these findings with neurophysiological evidence using multi-channel EEG and/or other devices.

Finally, we would like to consider how our findings can be translated into practical applications for rehabilitation. The present study showed that a conflict induced by the difference between the prior knowledge of color and the meaning of the presented stimulus prolonged the premotor time but did not change the COP onset in the Go/No-go task in healthy adults. However, it is not clear whether the same results would be obtained in the elderly. If the COP onset and toe-off are prolonged in the elderly, this task may allow us to assess age-related changes of cognitive functions related to traffic rules. Greater understanding of this field may contribute to the development of evaluation indices and rehabilitation methods for cognitive functions.

There are limitations that should be acknowledged in the study. First, the odd number of participants may have led to an imbalance between the Red Go/Green No-go and Green Go/Red No-go conditions. Additionally, although the tasks were performed in random order, the relatively small sample size may have introduced variability that was not fully controlled. These factors should be considered when interpreting the results. Second, color blindness was not tested, and while participants reported normal or corrected vision, undiagnosed color vision deficiencies could have influenced the results. Moreover, the cultural understanding of red and green was assumed but not explicitly tested.

## 5 Conclusion

The conflict between the prior knowledge of color about traffic rule and the meaning of stimulus color affected gait initiation. Specifically, the onset for the TA muscle activity, but not the onset of COP movement, was delayed in the Red Go/Green No-go than Green Go/Red No-go task. This finding suggests that the prior knowledge about traffic signal colors influences only the initial motor command during gait initiation in healthy adults.

## Data Availability

The raw data supporting the conclusions of this article will be made available by the authors, without undue reservation.
